# The millipede *Typhloglomeris caucasica* Golovatch, 1975 found epigeically (Diplopoda, Glomerida, Glomeridellidae)

**DOI:** 10.3897/BDJ.1.e981

**Published:** 2013-09-16

**Authors:** Sergei Golovatch, Yuri A. Chumachenko

**Affiliations:** † Russian Academy of Sciences, Moscow, Russia; ‡ Caucasian State Biosphere Nature Reserve, Research Department, Sovetskaya Street 187, Maikop, Republic of Adygeya, Russia

**Keywords:** Diplopod, *Typhloglomeris caucasica*, Caucasus, Sochi, cave, forest litter, new record

## Abstract

The millipede *Typhloglomeris caucasica* Golovatch, hitherto considered as a troglobite confined to several caves near Sochi, western Caucasus, Russia, is recorded epigeically in the same region, and is therefore a troglophile.

## Introduction

The small diplopod family Glomeridellidae contains only two valid genera: *Glomeridella* Brölemann, 1913, with 7-8 species ranging from France in the west, through the eastern Alps, to the Balkans in the east, and *Typhloglomeris* Verhoeff, 1898, with about 15 species, a few of which are presumed troglobites, from the Balkans, Caucasus, northwestern Iran, and Asia Minor (Golovatch 2003, Makarov et al. 2003). All *Glomeridella* and most *Typhloglomeris* species show distinctive colour patterns and are known to be epigean, but even among the few colourless representatives not all are troglobites. Thus, *Typhloglomeris fiumarana* Verhoeff, 1899 has been regarded as possibly a geobiont (Golovatch 1989, Golovatch 2003) because it was found in Croatia under stones, not in a cave. Similarly, *Typhloglomeris alba* (Golovatch, 1989), in which only the ocelli are pigmented, whereas the rest of the body is pallid, is clearly epigean in northwestern Anatolia, Turkey (Golovatch 1989). Only the following fully unpigmented congeners have hitherto been referred to as troglobites (Golovatch 1975, Golovatch 1989, Golovatch 2003, Makarov et al. 2003): *Typhloglomeris coeca* Verhoeff, 1898, from Croatia and Montenegro, *Typhloglomeris varunae* Makarov, Lučić, Tomić & Karaman, 2003, from Macedonia, *Typhloglomeris seuti* Makarov, Lučić, Tomić & Karaman, 2003, from Montenegro, and *Typhloglomeris caucasica* Golovatch, 1975, from near Sochi, western Caucasus, Russia. Furthermore, based on several morphological characters, Makarov et al. 2003 treat *Typhloglomeris caucasica* as representing a species group of its own, the *caucasica*-group.

## Taxon treatments

### Typhloglomeris caucasica

Golovatch, 1975

#### Materials

- **Type status:** Other material. **Occurrence:** recordedBy: Y. A. Chumachenko; individualCount: 1; sex: male; **Taxon:** kingdom: Animalia; phylum: Arthropoda; class: Diplopoda; order: Glomerida; family: Glomeridellidae; genus: Typhloglomeris; specificEpithet: caucasica; scientificNameAuthorship: Golovatch, 1975; **Location:** country: Russia; stateProvince: Sochi; verbatimLocality: Khosta, Caucasian Biosphere Nature Reserve; **Event:** samplingProtocol: pitfall trapping; eventDate: 14 July - 10 August 2006; habitat: Taxus & Buxus relict forest; **Record Level:** institutionCode: Zoological Museum, Moscow State University, Russia

#### Discussion

The recent discovery of *Typhloglomeris caucasica* in a pitfall trap in forest near Sochi indicates that the ecological status of this species is a troglophile, not a troglobite, when it occurs in caves in the same area. This record emphasizes the need to carefully collect near and outside caves when assessing the degree of cavernicoly of endogean animals.

## Supplementary Material

XML Treatment for Typhloglomeris caucasica
